# Differences in Genetic Diversity of Mammalian Tick-Borne Flaviviruses

**DOI:** 10.3390/v15020281

**Published:** 2023-01-19

**Authors:** Kassandra L. Carpio, Jill K. Thompson, Steven G. Widen, Jennifer K. Smith, Terry L. Juelich, David E. Clements, Alexander N. Freiberg, Alan D. T. Barrett

**Affiliations:** 1 Department of Biochemistry and Molecular Biology, University of Texas Medical Branch, Galveston, TX 77555, USA; 2 Department of Pathology, University of Texas Medical Branch, Galveston, TX 77555, USA; 3 Hawaii Biotech Inc., Honolulu, HI 96817, USA; 4 Sealy Institute for Vaccine Sciences, University of Texas Medical Branch, Galveston, TX 77555, USA

**Keywords:** genetic diversity, flavivirus, tick-borne viruses, Deer Tick virus, Langat virus, Powassan virus, tick-borne encephalitis virus, Kyasanur Forest Disease virus, Alkhurma hemorrhagic fever virus, Far Eastern tick-borne encephalitis virus, Omsk hemorrhagic fever virus

## Abstract

The genetic diversities of mammalian tick-borne flaviviruses are poorly understood. We used next-generation sequencing (NGS) to deep sequence different viruses and strains belonging to this group of flaviviruses, including Central European tick-borne encephalitis virus (TBEV-Eur), Far Eastern TBEV (TBEV-FE), Langat (LGTV), Powassan (POWV), Deer Tick (DTV), Kyasanur Forest Disease (KFDV), Alkhurma hemorrhagic fever (AHFV), and Omsk hemorrhagic fever (OHFV) viruses. DTV, AHFV, and KFDV had the lowest genetic diversity, while POWV strains LEIV-5530 and LB, OHFV, TBEV-Eur, and TBEV-FE had higher genetic diversities. These findings are compatible with the phylogenetic relationships between the viruses. For DTV and POWV, the amount of genetic diversity could be explained by the number of tick vector species and amplification hosts each virus can occupy, with low diversity DTV having a more limited vector and host pool, while POWV with higher genetic diversities has been isolated from different tick species and mammals. It is speculated that high genetic diversity may contribute to the survival of the virus as it encounters these different environments.

## 1. Introduction

The mammalian tick-borne flavivirus (MTBF) complex includes serologically and genetically related viruses within the genus *Flavivirus* of the family *Flaviviridae* that are transmitted by ticks [1,2]. Tick borne encephalitis virus (TBEV) is primarily spread by ticks in the genus *Ixodes*, and is usually transmitted to small mammals, such as rodents, that act as amplifying hosts [3]. While ticks are the primary vector for these viruses, they also act as hosts, and the viruses can be present throughout the tick life cycle. Humans are typically incidental hosts that are infected through a tick bite or by the consumption of unpasteurized milk of infected mammals [4].

Flaviviruses are single-stranded, positive-sense RNA viruses. Their genomes are approximately 11 kb in length and translated as a single polyprotein that encodes three structural proteins (capsid (C), pre-membrane (prM), and envelope (E) and seven non-structural proteins (NS1, NS2A, NS2B, NS3, NS4A, NS4B, and NS5) [5].

Members of the MTBF group include the TBE virus (TBEV), which has three subtypes: the Central European (TBEV-Eur, also known as the western subtype), the Far Eastern (TBEV-FE, also known as Russian Spring-Summer encephalitis), and the Siberian (TBEV-Sib), that are all neurotropic [2]. In addition, the complex includes Louping ill virus (LIV) and Powassan virus (POWV), which may cause encephalitis in mammals [3]. Deer Tick virus (DTV) is a genotype of POWV, and as the name implies, the virus is isolated from deer ticks, *Ixodes scapularis*. Omsk hemorrhagic fever virus (OHFV), Kyasanur Forest Disease virus (KFDV), and Alkhurma hemorrhagic fever virus (AHFV) also belong to the MTBF group, but typically cause hemorrhagic fever instead of encephalitis, and AHFV is considered a genotype of KFDV. The langat virus (LGTV) is not pathogenic in humans [3].

There have been a few studies characterizing the genetic diversity of different members of the MTBF group [6,7,8,9,10,11,12,13], but only a fraction of those used in deep sequencing techniques [14,15,16,17]. Overall, most studies have focused on TBEV and AHFV [9,18,19,20,21,22], whereas other members of the complex, such as OHFV, KFDV, LGTV, and POWV, have been poorly characterized. This paper reports on the genetic diversity of many members of the MTBF group using Next Generation Sequencing (NGS), and shows that there are differences between the viruses within the group.

## 2. Materials and Methods

Viruses and Cells. KFDV strain P9605, AHFV strain Zaki-1, OHFV strain Guriev, TBEV-Eur strain Hypr, and TBEV-FE strain Sofjin were obtained from the World Reference Center for Emerging Viruses and Arboviruses (WRCEVA, UTMB). Virus stocks used for sequencing were prepared in either BHK-SA or BHK-S cells (see Table 1 for details) and virus infectivity titers determined by plaque assay in BHK-SA cells using 0.8% tragacanth overlay in MEM, supplemented with 5% FBS and 1% penicillin/streptavidin. All virus stocks were prepared under biosafety level 4 (BSL4) conditions at the Robert E. Shope and Galveston National Laboratory (GNL) BSL4 laboratories (UTMB). Prior to the extraction of viral RNA and sequencing, virus stocks were inactivated by gamma irradiation (5.0 × 10^4^ Gy).

POWV/LEIV-5530, DTV/NPS001, and DTV/IPS-001 were also obtained from the WRCEVA and UTMB, while POWV/LB and LGTV were obtained from the Arbovirus Unit, London School of Hygiene and Tropical Medicine. Virus stocks were prepared as previously described [7,23].

Viral RNA isolation. Viral RNA was isolated using the QIAamp Viral RNA Mini Kit (Qiagen, Germantown, MD, USA) from cell culture supernatants.

Next Generation Sequencing. Next Generation Sequencing (NGS) was performed at the UTMB Sequencing Core using the Illumina NextSeq 550 instrument with a paired-end 75 base protocol. Sequencing libraries were prepared from 10 to 50 ng of viral RNA using the NEBNext Ultra II RNA kit (New England Biolabs, MA, USA), following the manufacturer’s protocol.

Data analysis. Reads were randomly down sampled to an average coverage of 1000 reads, and subjected to a post processing pipeline, as previously described [24]. Briefly, reads below a quality score of 30 were filtered out, and the remaining reads were mapped using bowtie2 with the “very-sensitive-local” parameter. Per the bowtie2 manual found online, the local parameter uses soft clipping at the ends of reads so the first and last few nucleotides may not participate in the alignment to maximize the alignment score, and the very sensitive parameter trades speed for accuracy in the multiseed alignment and allows 20 consecutive seed attempts, three re-seed attempts, and sets the number of mismatches allowed to 0 and the seed substring length to 20. Variant calling was completed using Lofreq (v 2.1.3.1) [25]. The consensus sequence of AHFV was aligned to Genbank JF416957.1, KFDV was aligned to Genbank JF416958.1, LGTV were aligned to Genbank AF253419.1, OHFV was aligned to Genbank AB507800.1, POWV was aligned to Genbank KT224351.1 for the LEIV-5530 strain and L06436.1 for the LB strain, DTV was aligned to Genbank HM440559.1, TBEV-FE was compared to Genbank JF819648.2, and TBEV-Eur was aligned to Genbank MT228627.1.

Statistical analysis. Statistical analysis was performed using GraphPad Prism (v 8.4.2). Shannon entropy was compared between viruses and within each strain. The Kruskal-Wallis test with Dunn’s multiple comparisons test was used to identify statistical significance in Shannon entropy, where *p* < 0.05 was considered statistically significant. 

Phylogenetic analysis. The sequences were aligned beginning with the polyprotein start codon and included all 10 genes. A phylogenetic analysis was performed using Clustal W aligned sequences beginning at the coding region. The neighbor-joining tree was constructed using the Jukes-Cantor algorithm in MEGA (version 11.0.13). The rates among variable sites were changed to a gamma distribution with a 0.48 parameter (also calculated in MEGA). Gaps or missing data were subjected to pairwise deletions, and all codon positions and non-coding sites were selected. A bootstrap analysis of 500 replications was performed, and the numbers on the tree represent the bootstrap values [9].

Reference strains were used for comparison. Eight viruses from the MTBF group were sequenced using NGS. This included multiple strains of some viruses and a different passage history for other viruses, as presented in Table 1. The viruses used in this study are referred to by their abbreviation shown in column 1 of Table 1. The consensus sequences of the virus genomes were compared to the published sequences of each strain designation noted on the virus stock tested. Since DTV/NPS001 and DTV/IPS-001 had no published sequences for the full virus strains available, they were compared to the sequence with the highest similarity using The National Center for Biotechnology Information (NCBI) BLAST, which was HM440559.1.

## 3. Results

Comparison of consensus sequences

The AHFV/Zaki-1, LGTV/TP21_I, and LGTV/TP21_II had no consensus coding changes compared to their published genomes. LGTV/TP21_I and LGTV/TP21_II had 100% identity compared to their respective reference genomes, while LGTV/TP21_III had one nonsynonymous difference in E-T156I, which was predicted to abolish a potential envelope protein N-linked glycosylation site since the motif from residues 154–156 follows the amino acid sequence N-X-S/T [26]. AHFV/Zaki-1 had one synonymous difference at NS3-V63 (Table 1). POWV/LB had one synonymous difference at NS4A-31 and one nonsynonymous difference at E-Y132H, while POWV/LEIV-5530 had one nonsynonymous difference in the E protein at E-H132Y. KFDV had one nonsynonymous difference at NS4A-T75A. OHFV/Guriev had two synonymous differences in NS2A and NS4A plus one nonsynonymous difference at NS3-V371I. Both sequences of DTV/IPS-001_1 and DTV/IPS-001_II shared the same 20 synonymous differences and four nonsynonymous differences from the published reference sequence. DTV/NPS001 had one additional synonymous mutation at NS3-455 compared to its closest BLAST match. The TBEV-Eur/Hypr sequence had 12 synonymous and six nonsynonymous differences, while TBEV-FE/Sofjin had 13 synonymous and eight nonsynonymous differences compared to the published sequences. Overall, AHFV/Zaki-1, KFDV/P9605, LGTV/TP21, OHFV/Guriev, and POWV/LIEV-5530 closely matched the published sequences, while DTV/IPS-001, DTV/NPS001, TBEV-Eur/Hypr, and TBEV-FE/Sofjin had additional differences but still shared at least 99% nucleotide identity compared to the published sequences (Table 1). 

**Figure 1:**
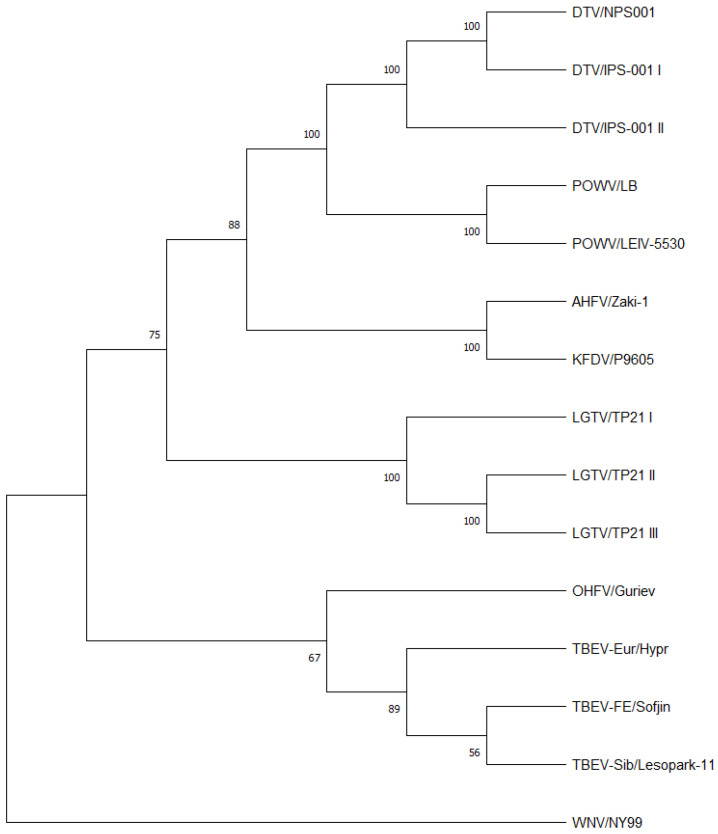
Phylogenetic analysis of the coding regions of listed viruses. The neighbor-joining tree was constructed using the Jukes-Cantor algorithm in MEGA 11.0.13. The numbers on the tree represent bootstrap values from 500 replications. Note all viral sequences were derived in this and previous studies except for TBEV-Sib Lesopark 11, which was taken from Genbank.

A phylogenetic tree was constructed from the consensus genome sequences of the MTBFs sequenced in this study, a TBEV-Sib sequence from Genbank [note the authors do not have a TBEV-Sib isolate available for these studies], and a West Nile virus strain NY99 previously sequenced in the author’s laboratory, as an outgroup (Figure 1). Overall, the MTBFs showed the expected relationship at the consensus genome level as reported in previous studies [9,11,27,28,29] with AHFV and KFDV closely related to each other (considered as genotypes), TBEV-FE, TBEV-Eur and OHFV closely related to each other, and POWV and DTV closely related to each other (considered as genotypes).

Genetic diversity within the viral RNA population

**Figure 2:**
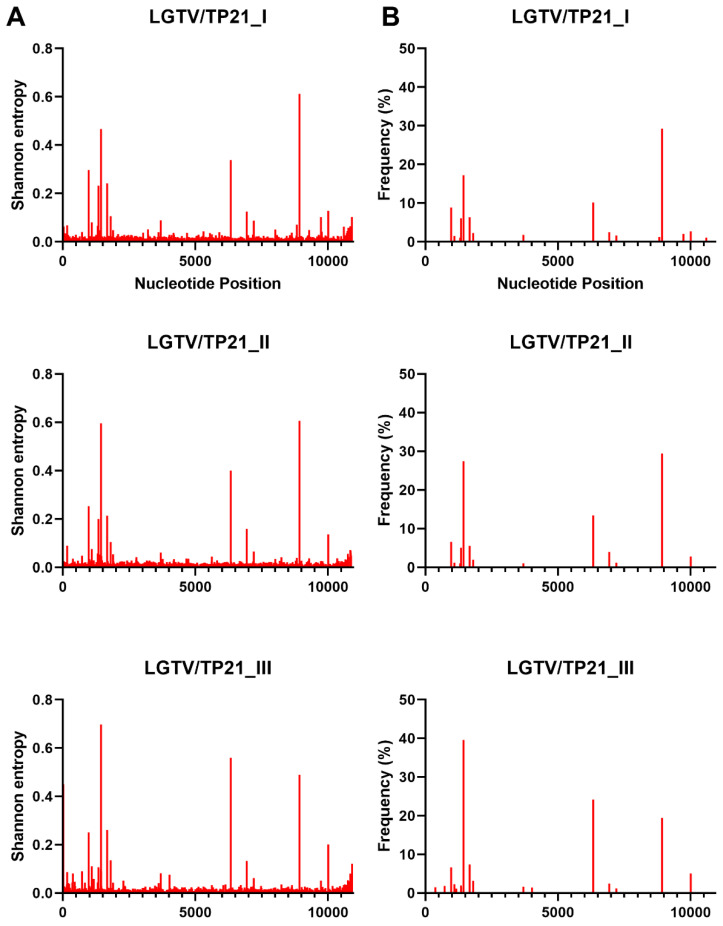
Shannon entropy (**A**) and SNVs (**B**) for each LGTV sample. See Table 1 for details of each sample and virus sample code.

**Figure 3:**
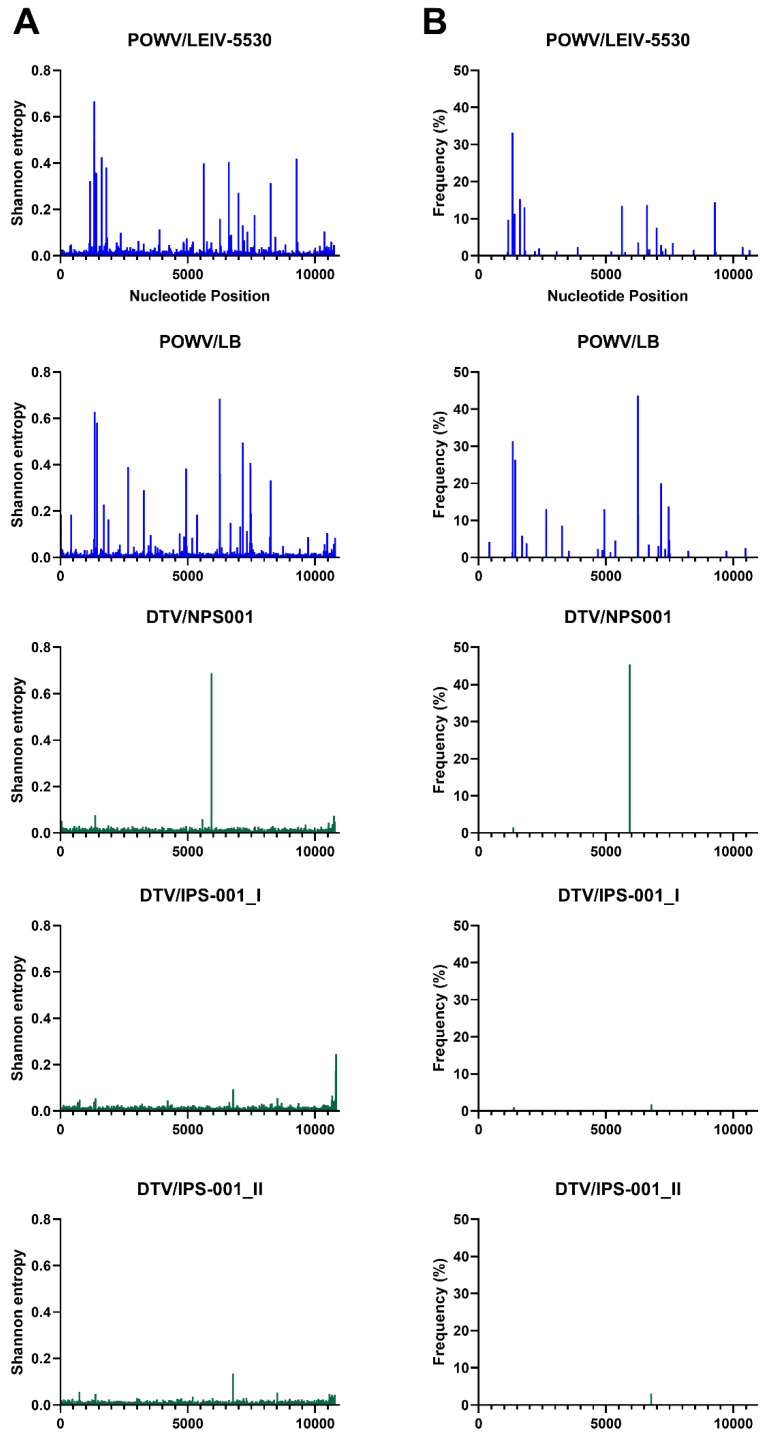
Shannon entropy (**A**) and SNVs (**B**) for each POWV and DTV sample. See Table 1 for details of each sample and virus sample code.

**Figure 4:**
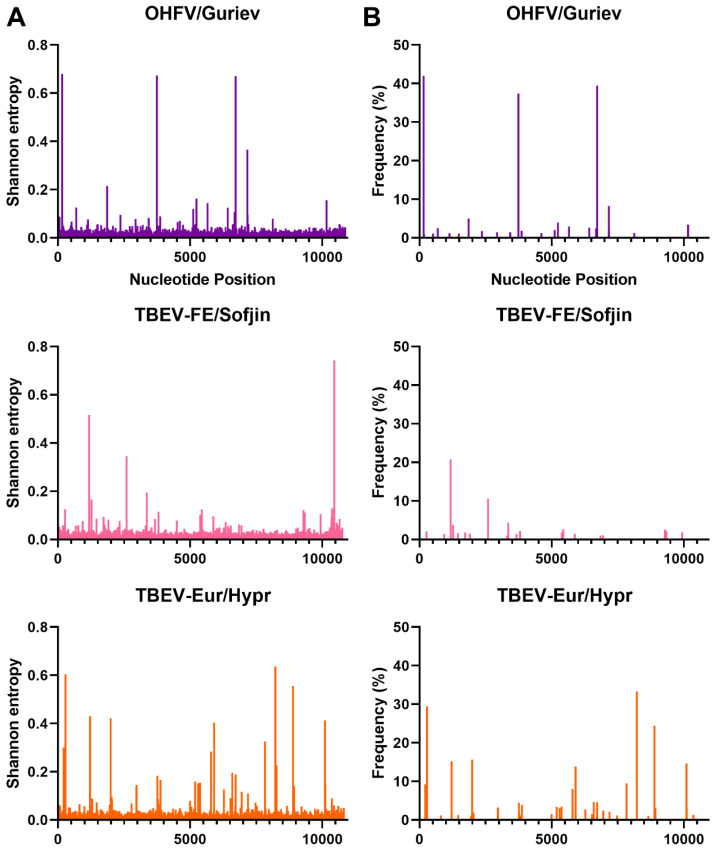
Shannon entropy (**A**) and SNVs (**B**) for OHF, TBE-FE, and TBE-Eur viruses. See Table 1 for details of each sample and virus sample code.

**Figure 5:**
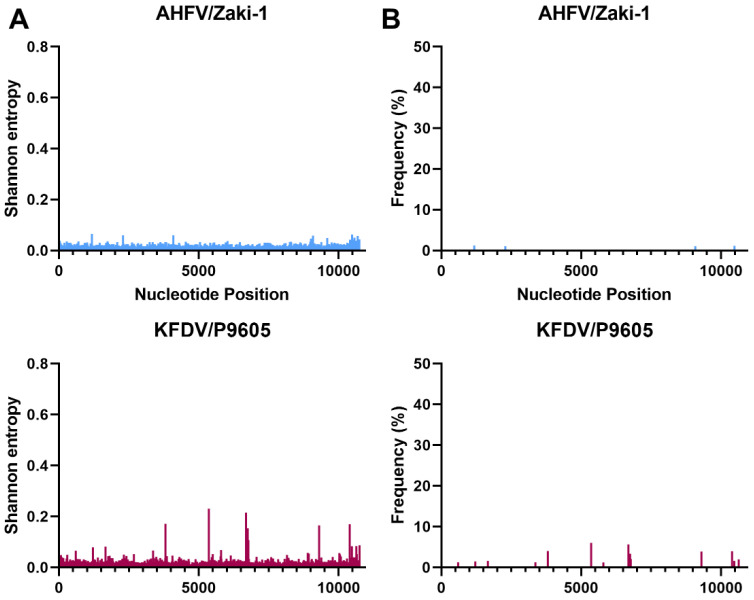
Shannon entropy (**A**) and SNVs (**B**) for AHF and KFD viruses. See Table 1 for details of each sample and virus sample code.

NGS was used to investigate the genetic diversity of each MTBF member in this study in terms of Shannon entropy and single nucleotide variants (SNVs) (Figure 2, Figure 3, Figure 4 and Figure 5). The depth of coverage of AHFV and KFDV were initially low, therefore, reads used in the analysis of this virus are a combination of three separate NGS runs. The first two NGS runs used genomic RNA that was extracted from the same ampoules on two separate occasions, while the third run used RNA from the same virus stock in a different vial. Each individual run had a low mean depth of coverage, but the Shannon entropy and SNVs were very similar for the three runs (data not shown), and when combined, they gave an average of about 1000 reads per base. In order to accurately compare the Shannon entropy and SNVs for each virus, the remaining viruses’ reads were randomly down-sampled to a mean of 1000 reads at each nucleotide position.

Interestingly, there were differences between the Shannon entropy of particular viruses. AHFV was different from every other virus in this study (*p* < 0.0001), and KFDV differed from most viruses except the two TBEV strains (*p* > 0.9999) (Figure 2 and Table S1). LGT, POW, and DT viruses were different compared to OHFV, TBEV-FE, TBEV-Eur, AHFV, and KFDV (*p* < 0.0001). LGTV/TP21_I differed from LGTV/TP21_II and LGTV/TP21_III (*p* = 0.0332 and 0.0014, respectively) (Figure 2). LGTV/TP21_I also differed from POWV/LB and DTV/IPS-001 (*p* = 0.0002 and 0.0026, respectively). The statistics are summarized in Table S1. Of the viruses used in this study, DTV/NPS001, the two DTV/IPS-001 isolates, KFDV/P9605, and the AHFV/Zaki-1 viruses had the lowest Shannon entropy, whereas TBEV-Eur/Hypr, TBEV-FE/Sofjin, and the two POWV strains had the highest Shannon entropy (Figure S1). 

In terms of SNVs, there was considerable variability between the viruses. POWV/LEIV-5530 had 32 SNVs ranging from 1.0–33%, and POWV/LB had 29 SNVs ranging from 1.1–44% (Figure 3). In comparison, DTV/NPS001 had three SNVs ranging from 1.4–45%, DTV/IPS-001_I had two SNVs ranging from 1.0–1.8%, and DTV/IPS-001_II had one SNV with 3.1% frequency. LGTV/TP21_I had 16 SNVs ranging from 1.1–29%, LGTV/TP21_II had 13 SNVs ranging from 1.0–29%, and LGTV/TP21_III had 19 SNVs ranging from 1.2–40% (Figure 2). TBEV-Eur/Hypr had 39 SNVs ranging from 1.0–33%, and TBEV-FE/Sofjin had 20 SNVs ranging from 1.0–21% (Figure 4). OHFV/Guriev had 24 SNVS ranging from 1.0–42%. KFDV/P9605 had 15 SNVs ranging from 1.2–6.0%, whereas AHFV/Zaki-1 had four SNVs ranging from 1.0–1.2% (Figure 5).

## 4. Discussion

This study has compared the genetic diversity of different members of the MTBF group by NGS. The analyses of the consensus sequences obtained in this study were very closely related to the genomic sequences reported in the literature and their genetic relationships, giving confidence to the analysis of genetic diversity (Figure 1). NGS revealed differences in genetic diversity between members of the TBE complex based on Shannon entropy and SNVs (summarized in Figure S1). Charrel et al. [9] analyzed AHFV, a genotype of KFDV, from infected patients in Saudi Arabia, and was the first group to identify the low genetic diversity seen in this virus. The low genetic diversity was observed regardless of time of year, symptoms of infected patients, route of infection, and environment where the patient lived [9]. Our results support these studies that suggest that AHFV has low genetic diversity since the Shannon entropy was low and there were only four SNVs greater than 1%, and none of them exceeded 1.2% frequency (Figure 5). Interestingly, KFDV has previously been described as having a low genetic diversity that was comparable to AHFV [14]. In our analysis, KFDV had approximately four times the number of SNVs as AHFV (Figure 5); however, our analysis is based only on one strain each of AHFV and KFDV. Nonetheless, if correct, the small increase in genetic diversity of KFDV compared to AHFV may contribute to the increase in morbidity and mortality when infection of the two viruses was compared in immunocompetent mice [30]. Although no SNVs identified in this study directly matched those under selection pressure for the previous 11 KFDV strains studied [14], one SNV was found at nucleotide position 6690 in 5.7% of the KFDV/P9605 population that falls directly between two sites they identified to be under selection pressure at amino acids 2186 and 2188 in the published study. This would cause a nonsynonymous amino acid change from an alanine to a threonine at amino acid 2187 (NS4A-T75A).

The results from the different POWV and DTV samples suggest that genetic diversity within each strain remains consistent within the constraints of the limited passage histories studied, but the LGTV/TP21 with an additional passage in LLC-MK-2 cells (LGTV/TP21_I) was statistically different from the other two LGTV/TP21 viruses sequenced. However, it is difficult to identify whether those additional passages caused the difference in genetic diversity or there are other factors involved. 

Despite DTV being a genotype of POWV (Figure 1), the DTV isolates examined had a more homogenous viral population compared to POWV/LEIV-5530 and POWV/LB, with the latter two strains having approximately 10 times the number of SNVs as DTV/NPS001. The divergence between POWV and DTV was thought to occur due to positive natural selection; however, they are antigenically indistinguishable from each other [7,10,31]. The POWV genotype differs from DTV in that POWV has two initiation codons for the C protein and cleavage sites of the polyprotein have one or two amino acid differences [27]. An explanation for the decreased genetic diversity in DTV, compared to POWV, may be that DTV is primarily transmitted by *I. scapularis* ticks only, while POWV has been isolated from *I. marxi*, *I. cookei*, and *I. spinipalpis* in North America [31,32], but this hypothesis would need additional studies to confirm. 

Interestingly, the DTV/IPS-001 and DTV/NPS001 strains share 24 of 25 mutations detected (Table 1), and the POWV/LEIV-5530 strain was determined to have 100% nucleotide identity with POWV/LB. Therefore, there is a possibility that, in both cases, the same virus strain has been given different names prior to their use in these studies. Cross-contamination of the virus stocks is considered very unlikely since they did not share any SNVs. DTV/IPS-001_II had one SNV at genome position 6777 with 3.1% frequency, DTV/NPS001 had three at nucleotides 5938, 1362, and 1367 with 45, 1.5, and 1.4% frequency, respectively, and DTV/IPS-001_I had two at 6783 and 1378 with 1.8 and 1.0% frequency, respectively.

In conclusion, these results provide one of the first reports on the differences and similarities of the genetic diversity across a wide range of MTBFs, some of which had not been previously subjected to deep sequencing. The results suggest that there may be a correlation between the phylogenetic grouping and the levels of genetic diversity observed in these viruses. However, additional studies would be needed to determine the genetic diversity inside and outside of the different hosts that tick-borne viruses occupy to further understand the evolution of the MTBFs [33].

## Figures and Tables

**Table 1:** Differences in coding region of sequenced viruses compared to those previously published in Genbank.

Virus Abbreviation	Virus Strain & Passage History	Comparison Accession #	% Nt Identity	Synonymous Changes		Nonsynonymous Changes	
				Nt	AA	Nt	AA
AHFV/Zaki-1	AHFV/Zaki-1 smb P1, smb P2, Vero P4, Vero E6 P2, BHK-SA P1	JF416957.1	99	4793	NS3-V63		
KFDV/P9605	KFDV/P9605 smb P9, Vero E6 P2, BHK-SA P1	JF416958.1	99			6690	NS4A-T75A
DTV/IPS-001_I	DTV/IPS-001 smb P1, Vero E6 P1	HM440559.1	99	189	C-P26	2650	NS1-A72T
				387	C-R92	4604	NS3-S9L
				789	prM-D111	4711	NS3-T45A
				1326	E-A127	8888	NS5-S414L
				1491	E-V182		
				2562	NS1-L42		
				2757	NS1-K107		
				2823	NS1-P129		
				4452	NS2B-E89		
				4770	NS3-E64		
				4779	NS3-V67		
				4881	NS3-A101		
				5322	NS3-F248		
				5895	NS3-E439		
				7455	NS4B-N188		
				8124	NS5-T159		
				8373	NS5-L242		
				9174	NS5-E509		
				9276	NS5-I543		
				9969	NS5-R774		
DTV/IPS-001_II	DTV/IPS-001 smb P3, BHK-21 P1	HM440559.1	99	189	C-P26	2650	NS1-A72T
				387	C-R92	4604	NS3-S9L
				789	prM-D111	4711	NS3-T45A
				1326	E-A127	8888	NS5-S414L
				1491	E-V182		
				2562	NS1-L42		
				2757	NS1-K107		
				2823	NS1-P129		
				4452	NS2B-E89		
				4770	NS3-E64		
				4779	NS3-V67		
				4881	NS3-A101		
				5322	NS3-F248		
				5895	NS3-E439		
				7455	NS4B-N188		
				8124	NS5-T159		
				8373	NS5-L242		
				9174	NS5-E509		
				9276	NS5-I543		
				9969	NS5-R774		
DTV/NPS001	DTV/NPS001/CT-390 smb P1, Vero P1	HM440559.1	99	189	C-P26	2650	NS1-A72T
				387	C-R92	4604	NS3-S9L
				789	prM-D111	4711	NS3-T45A
				1326	E-A127	8888	NS5-S414L
				1491	E-V182		
				2562	NS1-L42		
				2757	NS1-K107		
				2823	NS1-P129		
				4452	NS2B-E89		
				4770	NS3-E64		
				4779	NS3-V67		
				4881	NS3-A101		
				5322	NS3-F248		
				5895	NS3-E439		
				5943	NS3-T455		
				7455	NS4B-N188		
				8124	NS5-T159		
				8373	NS5-L242		
				9174	NS5-E509		
				9276	NS5-I543		
				9969	NS5-R774		
LGTV/TP21_I	LGTV/TP21 smb P8, Vero P1, LLC-MK-2 P1	AF253419.1	100				
LGTV/TP21_II	LGTV/TP21 smb P8, Vero P2	AF253419.1	100				
LGTV/TP21_III	LGTV/TP21 smb P9, Vero P1	AF253419.1	99			1437	E-T156I
OHFV/Guriev	OHFV/Guriev	AB507800.1	99	3741	NS2A-I75	5710	NS3-V371I
	BHK-21 P2, BHK-S P2			6721	NS4A-L87		
POWV/LB	POWV/LB LLC-MK-2 P2*	L06436.1	99	6537	NS4A-L31	1340	E-Y132H
POWV/LEIV-5530	POWV/LEIV-5530 smb P1, Vero P2, LLC-MK-2 P1	KT224351.1	99			1312	E-H132Y
TBEV-FE/Sofjin	TBEV-FE/Sofjin BHK-21 P1, BHK-SA P1	JF819648.2	99	1638	E-L223	869	M-M41I
				3398	NS1-C313	1170	E-N67D
				3812	NS2A-S99	1429	E-V153A
				3986	NS2A-L157	6097	NS3-T500I
				4484	NS2B-R93	6561	NS4A-Q34E
				5384	NS3-I262	7161	NS4B-S85A
				5654	NS3-D352	7533	NS4B-L209F
				6626	NS4A-V55	10263	NS5-S868A
				7874	NS5-Y70		
				8996	NS5-L444		
				9164	NS5-S500		
				10169	NS5-R835		
				10208	NS5-C848		
TBEV-Eur/Hypr	TBEV-Eur/Hypr Vero CCL-81 P1, BHK-SA P1	MT228627.1	99	1188	E-A72	283	C-A51T
				1623	E-R217	538	prM-A19S
				2103	E-P377	1976	E-T335R
				2142	E-H390	2426	E-G485V
				4416	NS2B-E70	3952	NS2A-T146A
				4524	NS2B-S106	8000	NS5-A111V
				5913	NS3-E438		
				6384	NS3-R595		
				6870	NS4A-F136		
				8415	NS5-G249		
				8844	NS5-R393		
				8893	NS5-L410		

Nt: nucleotide; AA: amino acid; smb: suckling mouse brain; nucleotide numbers correspond to the reference sequence numbering.

## Data Availability

Consensus sequences can be accessed via Genbank using accession numbers OP037813-OP037825. NGS datasets can be accessed via ArrayExpress using accession number E-MTAB-12112.

## Supplementary Materials

The following supporting information can be downloaded at: https://www.mdpi.com/article/10.3390/v15020281/s1, Table S1: Shannon entropy *p*-values and Figure S1: Summary of Shannon entropy and Single Nucleotide Variants for all sequenced viruses.
