# Supplementary material for: Differences in Genetic Diversity of Mammalian Tick-Borne Flaviviruses
Source: Viruses. 2023 Jan 19;15(2):281. doi: 10.3390/v15020281 (PMC9959157; doi:10.3390/v15020281)
Supplement: Supplementary file 1 [file viruses-15-00281-s001.zip › viruses-1972191-supplementary.pdf]

Supplementary Figure

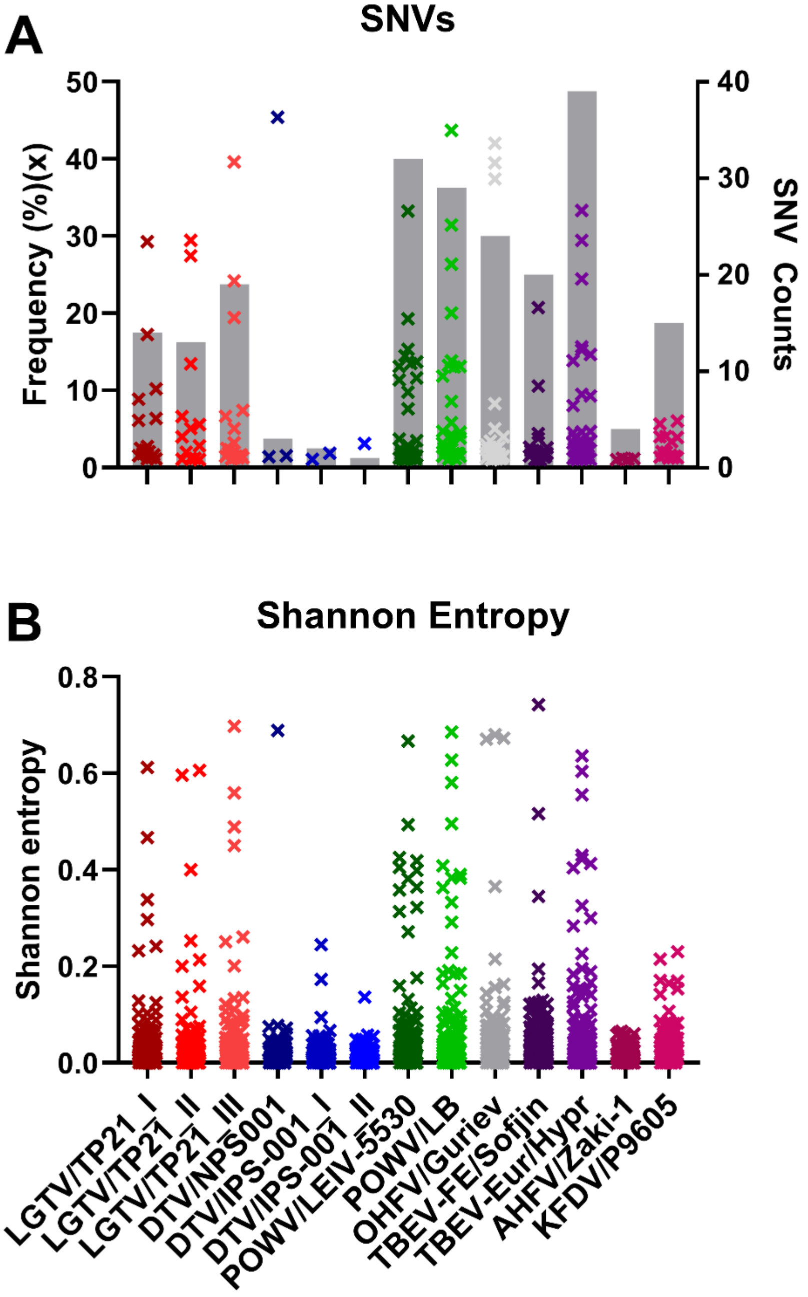

Supplementary Figure S1. Summary of SNVs (panel **A**), where each X represents a SNV, and their frequencies are on the left Y-axis. The gray bars represent the total counts of SNVs > 1% for each virus, and these correspond to the right Y-axis. Summary of Shannon entropy (panel **B**) of the different TBE complex viruses, where each X represents a nucleotide position with a non-zero Shannon entropy.

Supplementary Table S1

Table Supplementary S1. Shannon entropy p-values using Kruskall-Wallis test with Dunn's multiple comparisons.

	LGTV/TP21_I	LGTV/TP21_II	LGTV/TP21_III	POWV/LEIV-5530	DTV/NPS001	DTV/IPS-001_I	POWV/LB	DTV/IPS-001_II	OHFV/Guriev	TBEV-FE/Sofjin	TBEV-Eur/Hypr	AHFV/Zaki-1	KFDV/P9605
LGTV/TP21_I	-	0.0332	0.0014	0.1437	0.06	>0.9999	0.0002	0.0026	<0.0001	<0.0001	<0.0001	<0.0001	<0.0001
LGTV/TP21_II	0.0332	-	>0.9999	>0.9999	>0.9999	>0.9999	>0.9999	>0.9999	<0.0001	<0.0001	<0.0001	<0.0001	<0.0001
LGTV/TP21_III	0.0014	>0.9999	-	>0.9999	>0.9999	>0.9999	>0.9999	>0.9999	<0.0001	<0.0001	<0.0001	<0.0001	<0.0001
POWV/LEIV-5530	0.1437	>0.9999	>0.9999	-	>0.9999	>0.9999	>0.9999	>0.9999	<0.0001	<0.0001	<0.0001	<0.0001	<0.0001
DTV/NPS001	0.06	>0.9999	>0.9999	>0.9999	-	>0.9999	>0.9999	>0.9999	<0.0001	<0.0001	<0.0001	<0.0001	<0.0001
DTV/IPS-001_I	>0.9999	>0.9999	>0.9999	>0.9999	>0.9999	-	0.3196	>0.9999	<0.0001	<0.0001	<0.0001	<0.0001	<0.0001
POWV/LB	0.0002	>0.9999	>0.9999	>0.9999	>0.9999	0.3196	-	>0.9999	<0.0001	<0.0001	<0.0001	<0.0001	<0.0001
DTV/IPS-001_II	0.0026	>0.9999	>0.9999	>0.9999	>0.9999	>0.9999	>0.9999	-	<0.0001	<0.0001	<0.0001	<0.0001	<0.0001
OHFV/Guriev	<0.0001	<0.0001	<0.0001	<0.0001	<0.0001	<0.0001	<0.0001	<0.0001	-	>0.9999	0.5352	<0.0001	0.0003
TBEV-FE/Sofjin	<0.0001	<0.0001	<0.0001	<0.0001	<0.0001	<0.0001	<0.0001	<0.0001	>0.9999	-	>0.9999	<0.0001	>0.9999
TBEV-Eur/Hypr	<0.0001	<0.0001	<0.0001	<0.0001	<0.0001	<0.0001	<0.0001	<0.0001	0.5352	>0.9999	-	<0.0001	>0.9999
AHFV/Zaki-1	<0.0001	<0.0001	<0.0001	<0.0001	<0.0001	<0.0001	<0.0001	<0.0001	<0.0001	<0.0001	<0.0001	-	<0.0001
KFDV/P9605	<0.0001	<0.0001	<0.0001	<0.0001	<0.0001	<0.0001	<0.0001	<0.0001	0.0003	>0.9999	>0.9999	<0.0001	-
